# A configurable five-step workflow for high-throughput hyperspectral image analysis and vegetation index mapping

**DOI:** 10.1016/j.mex.2026.104162

**Published:** 2026-09-13

**Authors:** Jae Gyeong Jung, Kyuchan Kim, Jae Yeob Jeong, Jun Hyuck Yoon, Eun Ji Bae

**Affiliations:** a Forest Biomaterials Research Center, National Institute of Forest Science, Jinju, 52817, Republic of Korea; b Department of Biological Sciences, Chungnam National University, Daejeon, 34134, Republic of Korea

**Keywords:** Push-broom sensor, UMAP, DBSCAN, Random forest classifier, Frequency distribution

## Abstract

This workflow describes a structured approach for hyperspectral image analysis of push-broom camera data acquired under laboratory and field conditions. The workflow comprises five sequential steps: (1) ROI extraction from reflectance-corrected hyperspectral datacubes; (2) unsupervised spectral clustering via UMAP dimensionality reduction and DBSCAN; (3) supervised pixel-level classification using a Random Forest Classifier (RFC) with highlight mask generation; (4) computation of vegetation indices from configurable formula definitions with summary outputs; and (5) frequency distribution analysis across experimental treatments, including pairwise histogram overlay for visual comparison. Analysis parameters are configurable so that the workflow can be calibrated to specific imaging conditions. The workflow was demonstrated using wheat-kernel and turfgrass datasets spanning VNIR and SWIR spectral ranges under indoor and outdoor imaging conditions.

A five-step workflow with user-adjustable parameters adaptable to diverse hyperspectral imaging conditions.

An unsupervised–supervised spectral classification pipeline (UMAP → DBSCAN → Random Forest Classifier (RFC)) for pixel-level segmentation.

An integrated pipeline from ROI extraction to treatment-level frequency distribution comparison with numerical histogram output.

## Graphical abstract

**Figure:**
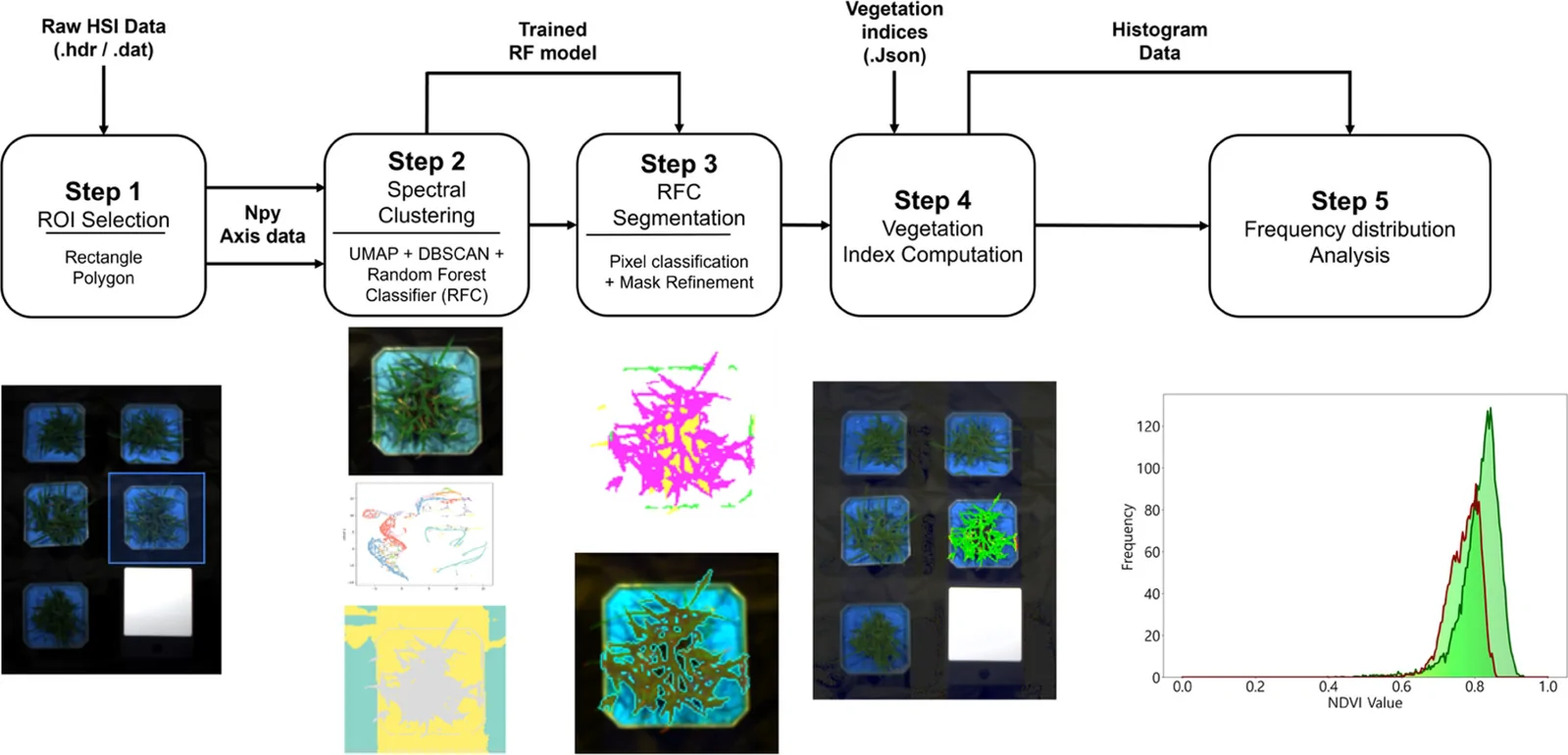

Specifications table

**Table:** 

**Subject area**	Agricultural and Biological Sciences
**More specific subject area**	Hyperspectral image analysis for plant phenotyping and vegetation monitoring using portable push-broom sensors
**Name of your method**	High-throughput hyperspectral image analysis workflow
**Name and reference of original method**	Uniform Manifold Approximation and Projection (UMAP) [1] Density-Based Spatial Clustering of Applications with Noise (DBSCAN) [2] Random Forest Classifier (RFC) [3]
**Resource availability**	Specim IQ hyperspectral camera (Specim, Spectral Imaging Ltd., Finland) [4] Software dependencies: listed in Supplementary Data SP1 ython source code: available as Supplementary Data (S1a-S5)

## Background

With the increasing use of hyperspectral imaging in agriculture, the amount of spectral information contained in a single image has grown substantially [5,6]. This increase in data volume has created a need for workflows that can process large hyperspectral datasets efficiently while remaining adaptable to different experimental conditions. Although a number of hyperspectral analysis software packages are available, many provide limited access to detailed parameter settings, which can restrict flexible use across experiments.

Portable hyperspectral imaging systems are widely used for specimen-level measurements because they provide high spatial and spectral resolution over relatively small imaging areas [7]. In contrast, large-area imaging systems often involve a trade-off between coverage and spatial resolution, which can lead to spectral mixing within individual pixels and complicate per-pixel analysis [8]. Most current hyperspectral imaging systems use push-broom acquisition, which generally provides higher spectral resolution than snapshot methods [9]. However, line-by-line acquisition requires additional time to capture a complete image, and multiple treatment groups or replications are therefore often arranged within a single image to improve acquisition efficiency. The resulting increase in spectral information also complicates the extraction of regions of interest (ROIs). Conventional methods can often separate vegetation from non-vegetation backgrounds [10], but separating spectrally similar plant materials that differ mainly by treatment condition remains more challenging. In addition, portable hyperspectral instruments are used across a wide range of laboratory and field conditions, making it difficult to define a single analysis method for all situations. Within an individual experiment or laboratory setting, however, imaging conditions are often sufficiently consistent to support repeated analysis once parameters have been calibrated [11].

The workflow therefore exposes clustering thresholds, classification depth, and vegetation-index definitions as adjustable settings. Once calibrated for a given imaging setup, these settings can be reused for images acquired under comparable conditions with minimal additional adjustment.

## Method details

The workflow consists of five sequential steps, with each output used in the next step. Multiple ROIs can be extracted from one datacube and analyzed in sequence. The functions do not run ROI batches automatically or concurrently. Model reuse is described in Step 2.5. Table 1 summarizes the main input-output flow for each stage.

**Table 1:** Concise overview of the five-step workflow.

Step	Main operation (code)	Input → main output
1	Broad manual ROI cropping (S1a/S1b with S1c)	Reflectance-corrected cube + broad user coordinates/vertices → ROI array + recorded coordinates
2	Model fitting (S2a-S2c)	ROI → UMAP embedding → DBSCAN labels → prediction map + fitted scaler/RFC
3	RFC application and overlay (S3)	Compatible ROI + saved scaler/RFC → prediction map + highlight overlay + caller-created filtered ROI
4	Vegetation-index analysis (S4a-S4c)	Filtered/original ROI + wavelengths/formula → index overlay + raw 250-bin counts
5	Frequency comparison (S5)	Paired frequency arrays → overlaid comparison figure

The compact functions return objects in memory. When persistent files are required, the calling script saves arrays (.npy), the scaler/RFC (joblib), and images or figures (.png); file serialization and iteration over multiple ROIs are not automated by the supplied functions.

### Prerequisites

#### Hardware requirements

**-** Tested Workstation: AMD Ryzen 5 3600 CPU (6 cores and 12 logical processors), 32 GB RAM, Windows. An NVIDIA RTX 3060 was present, but the supplied analysis functions are CPU-based and do not require GPU acceleration.
- Push-broom hyperspectral camera producing ENVI-format output (.hdr header + .dat/.raw binary data).

Table 2 reports rounded sums of warmed, stage-specific execution logs for fresh UMAP fitting, DBSCAN, and RFC in the original application, including plot generation. Initial ROI loading and user interaction are excluded. These system-dependent values are not end-to-end workflow or direct Step 3 inference times. After a model has been fitted to one ROI, spectrally comparable ROIs can proceed directly to Step 3 as described in Step 2.5.

**Table 2:** Representative ROI payloads and logged Step 2 times on the tested workstation.

Representative ROI	ROI shape	File size	Logged Step 2 time
NaCl turfgrass	127 × 127 × 204 (VNIR)	12.6 MiB	14.6 s
Traffic-test turfgrass	254 × 264 × 204 (VNIR)	52.2 MiB	64.8 s
Wheat kernels	366 × 280 × 288 (SWIR)	112.6 MiB	99.3 s

#### Software requirements

**-** Python 3.10–3.12 with scientific computing packages (see Supplementary Data SP1 for the full dependency list)

#### Data preparation

**-** ENVI-format HSI files (.hdr header and corresponding binary data file).
**-** The .hdr file must contain wavelength metadata for automatic band mapping.
- The supplied functions expect a reflectance-corrected cube arranged as height × width × bands. For the demonstration datasets, dark- and white-reference correction was completed before Step 1.

Step 1: Region-of-Interest Selection

1.1 Load the target HSI file

The target hyperspectral datacube is loaded for ROI selection. Wavelength metadata in the .hdr file are used to generate a pseudo-RGB preview. For VNIR data, bands closest to 650 nm, 550 nm, and 450 nm are assigned to red, green, and blue, respectively. For the SWIR sensor used, bands near 1134, 1335, and 1657 nm are assigned in the same order. The SWIR selection is sensor-specific and is used only for display and ROI delineation.

1.2 Define an ROI using one of two delineation methods:

An ROI may be delineated using one of two methods:

Rectangular ROI: a bounding box enclosing the target specimen.

Polygonal ROI: a custom boundary defined by user-placed vertices for irregularly shaped specimens.

**Fig. 1:**
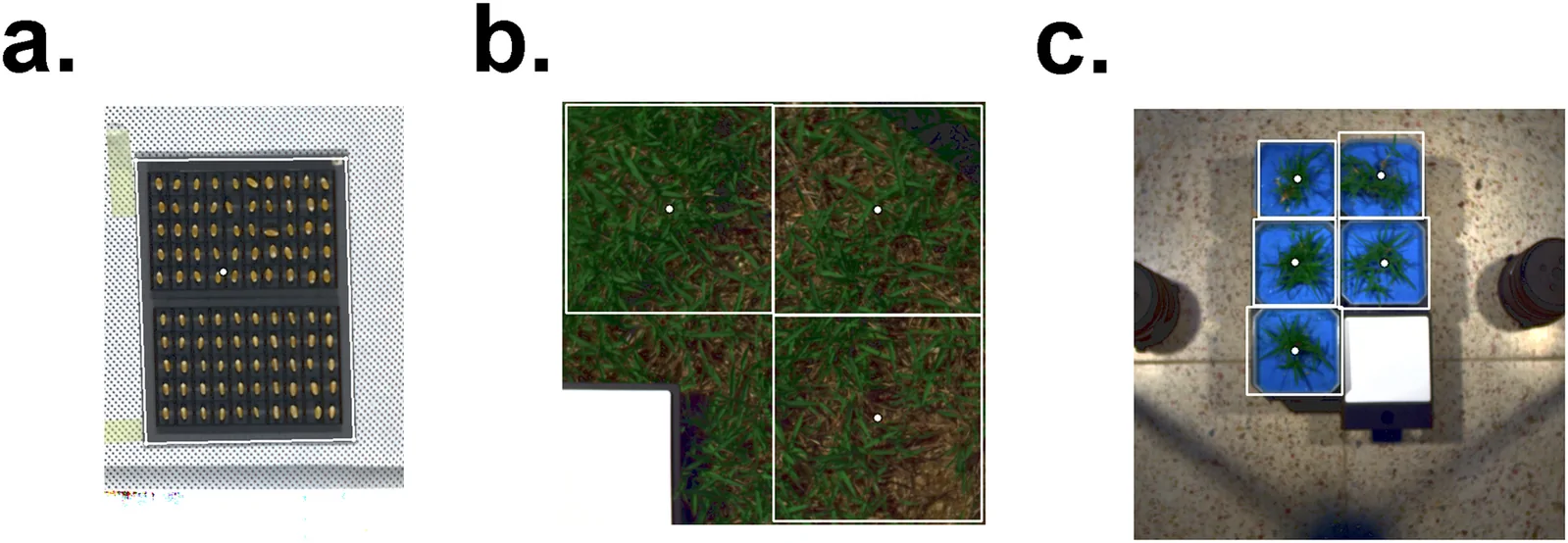
Representative ROI selections for the three specimens used for method validation. White boxes indicate user-selected ROIs: (a) wheat kernels (SWIR, indoor), (b) Turfgrass Sample A (VNIR, outdoor), and (c) Turfgrass Sample B (VNIR, indoor).

Representative ROI selections for the three demonstration datasets are shown in Fig. 1a–c, where white outlines indicate the manually selected ROIs.

Use rectangular ROIs for regularly arranged specimens and polygonal ROIs to exclude background around irregular targets. Because the ROI determines which spectra enter Step 2, apply the same drawing rule to treatments compared within an experiment.

As illustrated by the white outlines in Fig. 1a–c, Step 1 is a broad ROI-cropping step, not pixel-accurate manual segmentation of vegetation. The user only needs to draw a rectangular or polygonal ROI that fully encloses the target specimen; some surrounding background may be included because pixel-level separation is performed in Steps 2 and 3. Accordingly, defining an ROI is a straightforward coarse-cropping task. Excessive unrelated background should nevertheless be avoided.

1.3 Save the extracted region

S1a and S1b return the extracted ROI, and S1c returns its coordinates. Save these outputs as NumPy arrays when required.

1.4 Repeat for each sample

Steps 1.2–1.3 may be repeated to define several ROIs within one image. This reduces the number of pixels passed to Step 2 and allows treatment units in the same acquisition to be analyzed independently (Fig. 1a–c). Supplementary code for rectangular ROI extraction (S1a), polygonal ROI extraction (S1b), and ROI coordinate recording (S1c) is provided as Supplementary Data. The saved example ROI, ROI coordinates, and wavelength vector are provided in S7_02–S7_04; full filenames are listed in S7_01_README_matched_reference_set.txt.

Step 2: Spectral Clustering and Classification

2.1 Load the ROI data

Load one of the .npy files generated in Step 1.

2.2 Configure the spectral band range

Before running S2a, slice the ROI along the band axis if noisy or uninformative spectral extremes need to be removed.

2.3 Execute the analysis pipeline

The analysis proceeds in three stages:

**UMAP Dimensionality Reduction:** S2a reshapes the ROI into a pixel-by-band matrix, applies min-max scaling, and projects the spectra onto two dimensions using UMAP (n_neighbors = 15, min_dist = 0.1, Euclidean metric). These settings match the umap-learn defaults used in this study. A user-defined random seed supports repeatable embeddings. S2a returns the embedding; any caching is handled by the calling script. Table 3 summarizes the adjustment guidance.

**Table 3:** Parameter-adjustment guide.

Parameter	Value used	Diagnostic sign	Adjustment direction
UMAP n_neighbors	15	Local structure lost / embedding fragmented	Decrease / increase
DBSCAN eps	0.2	Distinct groups merge / one group fragments	Decrease / increase
DBSCAN min_samples	9	Small valid group becomes noise / isolated groups appear	Decrease / increase
RFC max_depth	2	RFC underfit DBSCAN-derived labels / map becomes patchy	Increase / decrease
RFC probability threshold	0.10	Background leakage / valid target pixels omitted	Increase / decrease

**Fig. 2:**
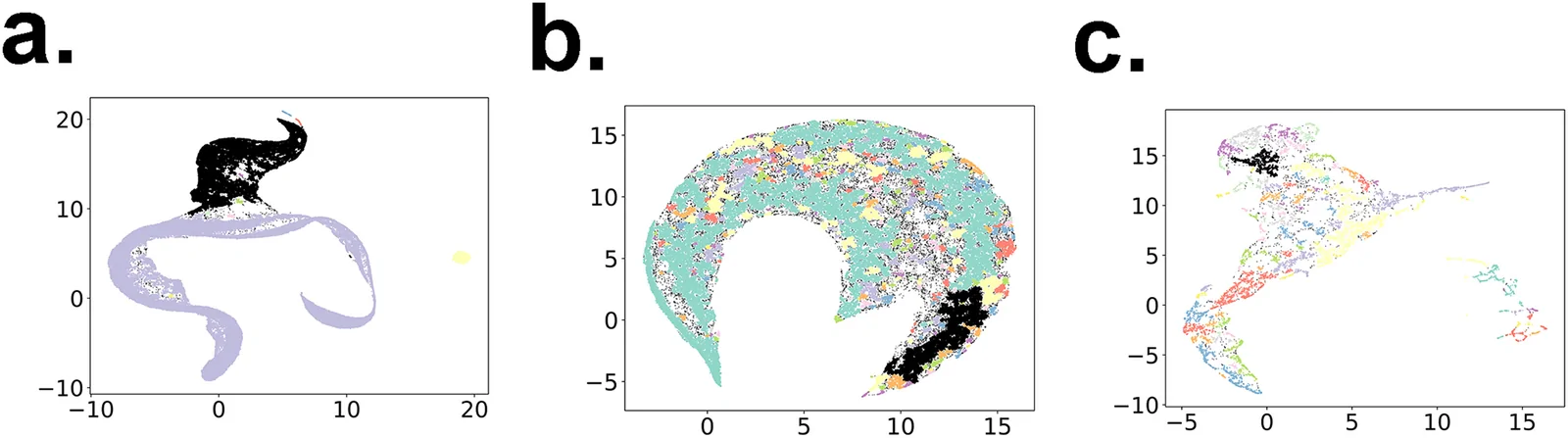
Representative UMAP embeddings colored by DBSCAN cluster labels for the selected ROIs: (a) wheat kernels, (b) Turfgrass Sample A, and (c) Turfgrass Sample B. Black-highlighted points indicate the target cluster selected for subsequent processing.

**DBSCAN Clustering:** DBSCAN is applied to the UMAP embedding using eps = 0.2 and min_samples = 20 by default in S2b; the matched S6 example uses min_samples = 9 (Table 3). S2b shifts the returned labels so that DBSCAN noise becomes class 0; class 0 does not necessarily represent physical background. The highlighted cluster is selected only as the downstream target, while the RFC is trained on all DBSCAN-derived labels (Fig. 2a–c). Adjustment cues are given in Table 3.

**Fig. 3:**
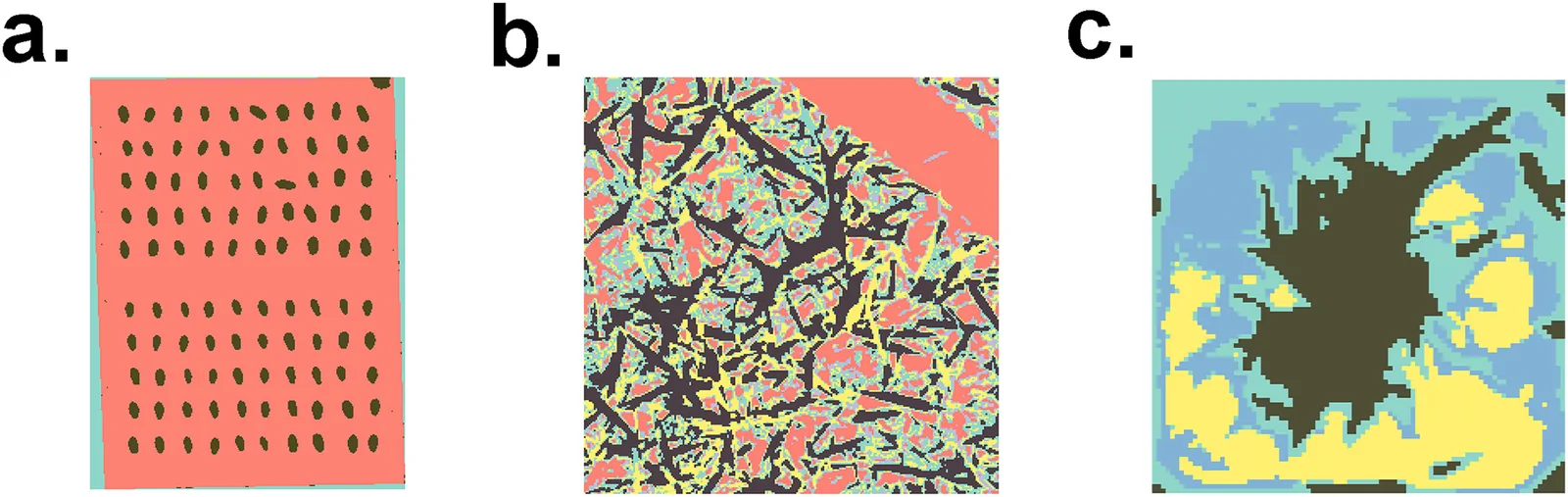
Representative RFC maps for the selected ROIs: (a) wheat kernels, (b) Turfgrass Sample A, and (c) Turfgrass Sample B. Black regions indicate the target class retained for subsequent processing.

**Random Forest Classifier (RFC):** S2c applies a separate min-max scaler and trains an RFC on all DBSCAN-derived pixel labels. The classifier uses 100 trees, the entropy criterion, min_samples_split = 10, and min_samples_leaf = 5. The supplied script uses a maximum depth of 2 (max_depth = 2) and a probability threshold of 0.1. S2c returns the prediction map, fitted scaler, and classifier. Save the scaler and classifier for Step 3 when model reuse is required (Fig. 3a–c). Table 3 gives the adjustment guidance.

2.4 Generate visualizations

In the original application, pseudo-RGB images, UMAP scatter plots, DBSCAN cluster maps, and RFC prediction maps were generated for rapid assessment of clustering and classification outputs.

2.5 Retain the fitted model

After fitting one ROI, save the RFC and scaler returned by S2c. They can be applied to other spectrally comparable ROIs from the same experiment when the sensor, selected bands, illumination, reflectance correction, and preprocessing remain unchanged. These ROIs can begin at Step 3 without repeating UMAP, DBSCAN, or RFC fitting, so the Step 2 times in Table 2 are not incurred for each ROI. Check the transferred segmentation before routine use.

**Model reuse versus refitting:** Reuse the saved scaler and RFC and proceed to Step 3 only when the sensor, retained band number and order, illumination, reflectance correction, preprocessing, expected spectral characteristics, and input feature dimension remain compatible. If any of these conditions changes, or if the transferred segmentation is unsatisfactory, repeat Step 2 on a representative ROI and save a newly fitted scaler and RFC. UMAP and DBSCAN are not repeated for compatible ROIs. Common acquisition changes and the corresponding recommended actions are summarized in Table 4.

**Table 4:** Common acquisition changes and recommended actions for model reuse or refitting.

Acquisition condition or change	Recommended user action
Comparable ROIs acquired with the same sensor, retained bands, illumination, reflectance correction, and preprocessing	Reuse the saved scaler and RFC in Step 3 when spectral characteristics remain comparable. Check the transferred segmentation before routine use.
Change in sensor, retained band number or order, or spectral sampling	Prepare a representative ROI with the required band configuration and repeat Step 2. Inspect the DBSCAN result, adjust its settings as needed, and save a newly fitted scaler and RFC.
Change in illumination, reflectance correction, or preprocessing	Repeat Step 2 using a representative ROI from the new conditions. Adjust DBSCAN settings as needed and fit the RFC to the resulting labels before applying it to further ROIs.
Change in image quality or expected spectral characteristics, or unsatisfactory transferred segmentation	Inspect the input ROI and repeat Step 2 on a representative ROI. Check the DBSCAN clusters and reconstructed spatial mask, adjust settings as needed, and refit the scaler and RFC.

Supplementary code for UMAP dimensionality reduction (S2a), DBSCAN clustering (S2b), and Random Forest Classifier (S2c) is provided as Supplementary Data. The S6 configuration and caller connect these functions to the matched UMAP/DBSCAN outputs (S7_05–S7_06) and fitted scaler/RFC (S7_08–S7_09).

Use the settings in Steps 2 and 3 as initial values. Change one parameter at a time, inspect both the embedding and reconstructed spatial mask, and check the result on another representative ROI. Recalibrate after changes in the sensor, spectral sampling, reflectance calibration, illumination, image quality, or expected spectral heterogeneity. Otherwise, retain the settings within the same acquisition batch.

Table 3 lists the principal values used in the matched S6 representative run. The UMAP random seed used for the reference run is 5000 and is recorded in S6. The corresponding workflow locations are UMAP (Step 2.3, S2a), DBSCAN (Step 2.3, S2b), RFC depth (Step 2.3, S2c), and the RFC probability threshold (Steps 2.3 and 3.3, S2c and S3).

Refitting in Step 2 regenerates the UMAP embedding for the representative ROI, checks DBSCAN clustering (adjusting eps and min_samples as needed), and fits the scaler and RFC using the DBSCAN-derived labels. UMAP and RFC settings can initially remain unchanged; further adjustment guidance is provided in Table 3. Select the target class label(s) again after refitting (Step 3.5).

**Step 3: Pixel-Level RFC Segmentation and Overlay**

3.1 Load the saved model

The RFC model and corresponding data scaler saved in Step 2 are loaded.

3.2 Load the target ROI

A target .npy file is loaded. The ROI data are prepared to match the feature dimensions expected by the trained model.

3.3 Apply the RFC

Each pixel is assigned a class label based on its spectral signature, producing a per-pixel classification result. Pixels whose maximum class probability falls below a threshold (0.1) are assigned to noise (label 0). See Table 3 for the corresponding threshold-adjustment guidance.

3.4 Generate a highlight overlay

A color-coded highlight mask is generated by combining two independent masks:

• **RFC mask:** pixels belonging to specified class labels.
• **RGB-similarity mask:** pixels whose pseudo-RGB values fall within a per-channel tolerance (default 0.10) of a target color.

**Fig. 4:**
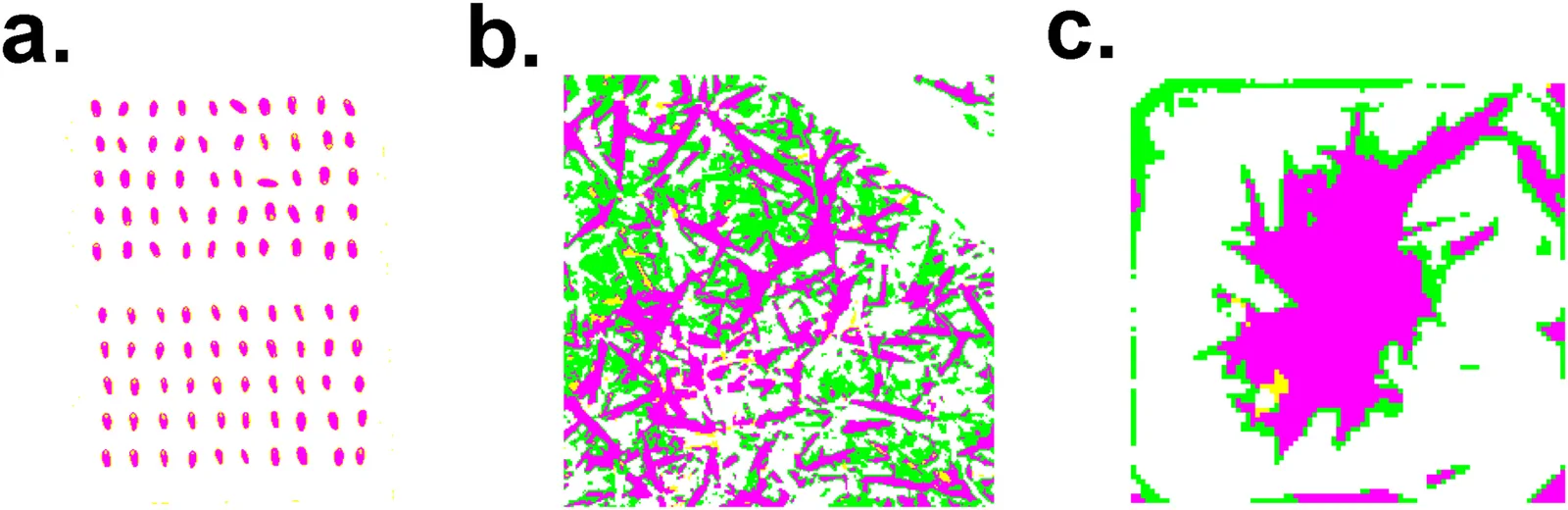
Representative color-coded highlight masks generated from RFC-based and RGB-similarity selection: (a) wheat kernels, (b) Turfgrass Sample A, and (c) Turfgrass Sample B. Yellow indicates RFC-selected only, green indicates RGB-similar only, and magenta indicates their intersection.

The resulting mask color-codes three regions: RFC-selected only (yellow), RGB-similar only (green), and their intersection (magenta) (Fig. 4a–c). This color-coded mask may also be overlaid semi-transparently on the pseudo-RGB image for visual assessment.

3.5 Return and save the segmentation result

S3 returns the prediction map. After the target RFC class label(s) have been selected by inspecting the reconstructed mask, the caller creates the filtered ROI as follows: mask = np.isin(rf_predictions, selected_labels); filtered_roi = np.where(mask[..., None], roi, 0). This retains the selected plant-associated pixels and sets all other pixels to zero before vegetation-index computation in Step 4. RFC/DBSCAN class identifiers are run-specific and must be selected again after refitting.

Supplementary code for RFC segmentation and overlay (S3) is provided as Supplementary Data. The matched prediction map is provided in S7_07, and the pseudo-RGB image, overlays, and filtered ROI are provided in S7_10–S7_13.

Step 4: Vegetation Index Computation

This step computes wavelength-based vegetation indices and generates summary outputs. In the original application used for the demonstrations, index formulas, target wavelengths, and display ranges were read from an external JSON configuration file. In the reduced S4a and S4c functions supplied with this article, the selected formula, wavelength vector, and ranges are passed directly by the caller.

A matched caller-side verification example is provided as Supplementary Data S6, comprising S6_workflow_config_example.json and the short S6_run_representative_example.py caller. Supplementary Data S7 contains the supplied ROI, ROI-coordinate and wavelength NPY files together with intermediate and final outputs generated from that same configuration and caller. The compact functions do not read the JSON file directly; the caller passes the exposed values and invokes the supplied functions. The tested package versions recorded in S6 are informational and exact version matching is not required. This compact single-ROI reference starts from the saved Step 1 ROI; S4b requires the full source datacube and S5 requires a second experimental ROI, so those optional stages are not duplicated in S7.

A representative entry used by the original application was structured as follows:

{

"NDVI":{

"formula":"(r800-r680)/(r800+r680)",

"wavelengths":{"NIR":800,"Red":680},

"range":{"min":0.0,"max":1.0}

}

}

In this notation, r{wavelength} denotes the reflectance at the specified wavelength in nanometers.

4.1 Apply an index formula to an ROI

Apply the selected formula to a filtered ROI from Step 3 or, when appropriate, to the original ROI from Step 1. The caller repeats this operation for additional ROI files.

4.2 Map spectral bands to target wavelengths

For each ROI, spectral bands are matched to target wavelengths using the supplied wavelength metadata. Users applying the workflow to another sensor must verify that every requested wavelength lies within the measured spectral range.

4.3 Generate visualization outputs

**Fig. 5:**
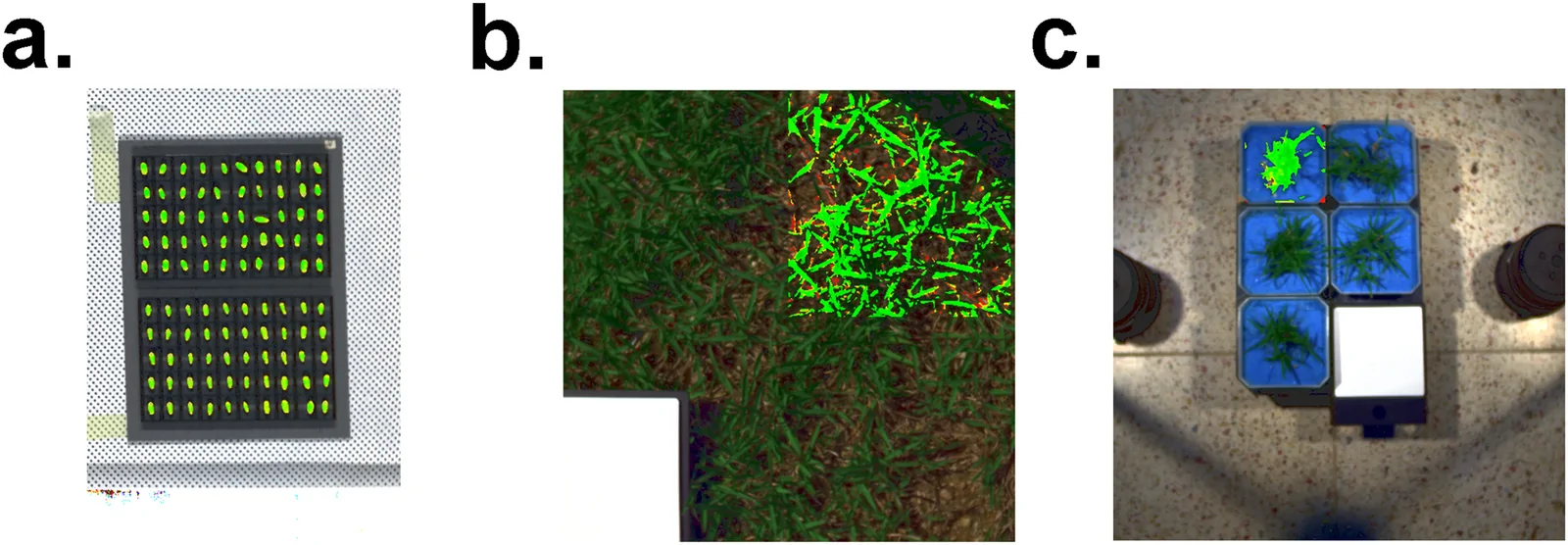
Representative vegetation index overlays on pseudo-RGB images for the three validation specimens: (a) wheat kernels (NDMI), (b) Turfgrass Sample A (NDVI), and (c) Turfgrass Sample B (NDVI).

**Fig. 6:**
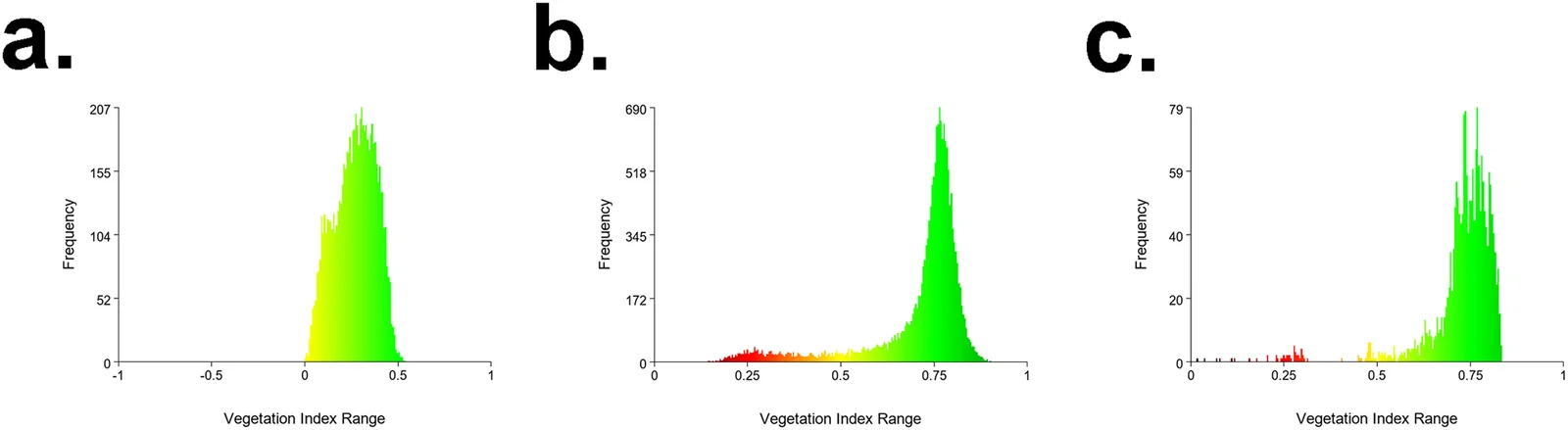
Representative 250-bin histograms of vegetation index values corresponding to Fig. 5: (a) wheat kernels (NDMI), (b) Turfgrass Sample A (NDVI), and (c) Turfgrass Sample B (NDVI).

Vegetation-index maps are generated from the computed values (Fig. 5a–c). The supplied function also returns raw 250-bin counts of finite, non-zero index values after clipping to the configured range (Fig. 6a–c); these counts are not normalized to proportions.

4.4 Repeat for additional ROI files

Repeat the same computation for additional ROI files using the same formula, range, and bin definition.

Supplementary code for vegetation index overlay (S4a), embedding the overlay on the original image (S4b), and vegetation index histogram (S4c) is provided as Supplementary Data. The matched S4a/S4c outputs are provided in S7_14–S7_16; S4b additionally requires the full source datacube.

**Step 5: Frequency Distribution Analysis**

5.1 Load frequency histograms

Raw frequency counts generated in Step 4 are loaded for comparison across treatments or measurement dates.

5.2 Pairwise treatment comparison

**Fig. 7:**
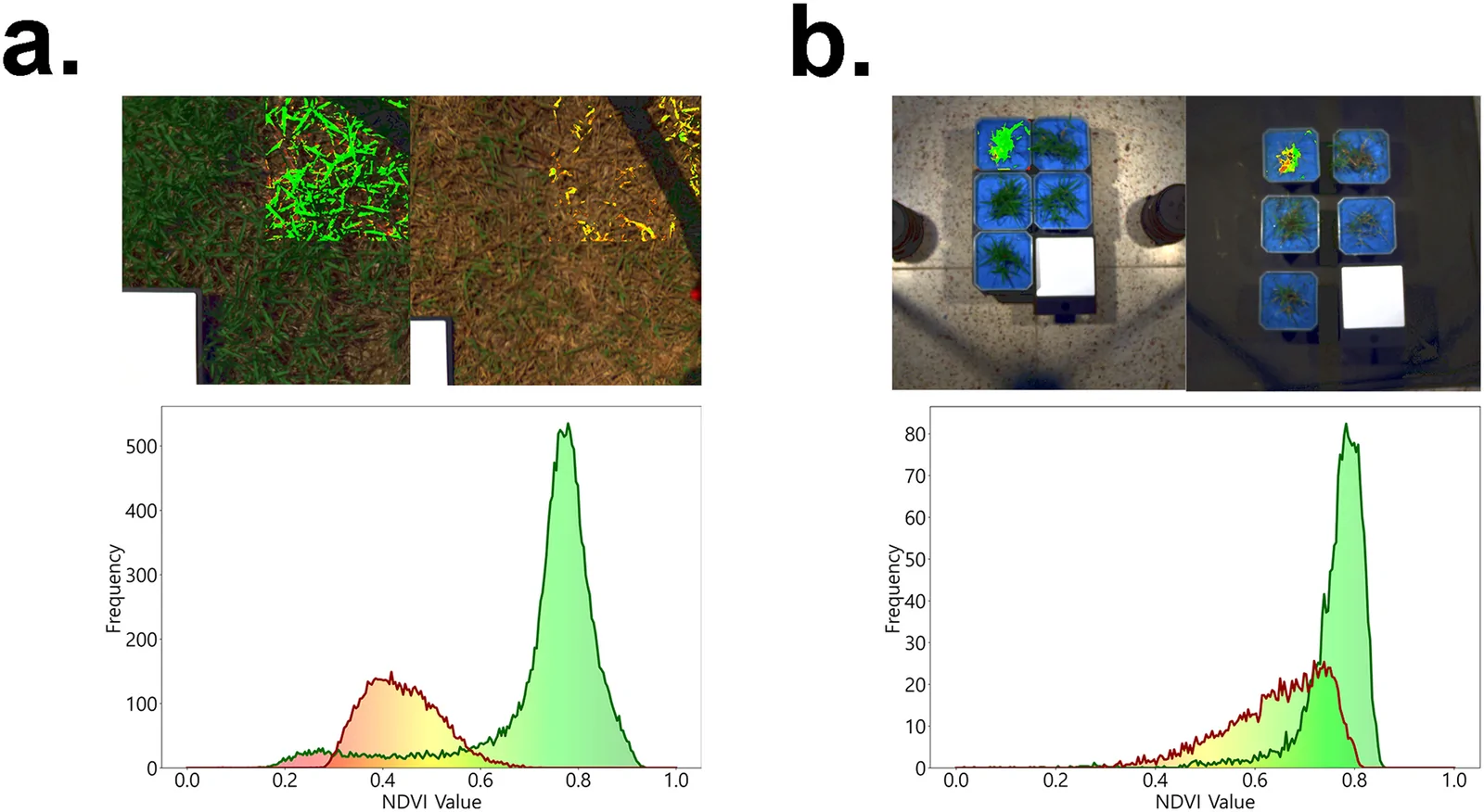
Representative pairwise comparisons of NDVI frequency distributions across measurement dates for (a) Turfgrass Sample A and (b) Turfgrass Sample B. Processed images from two time points are shown above, and the corresponding overlaid histograms are shown below.

For each vegetation index, paired raw-frequency histograms can be overlaid to examine distribution shifts across treatments or dates (Fig. 7a–b). The outputs represent pixel counts and are not interpreted as proportions unless normalization is performed separately by the caller.

Supplementary code for histogram comparison (S5) is provided as Supplementary Data. S5 uses paired histogram-frequency arrays from Step 4 and is not included in the single-ROI S6/S7 example.

After reflectance correction, run S1a or S1b with S1c, followed by S2a–S2c. Save the trained model and scaler before running S3, S4a–S4c, and S5. The user script handles data loading, file saving, and iteration over multiple ROIs.

## Method validation

The workflow was applied to three datasets covering VNIR and SWIR spectral ranges and both indoor and outdoor imaging conditions.

**Wheat kernels (SWIR, Indoor):** The datacube was corrected to reflectance using dark and white references before analysis. A single polygonal ROI enclosed 100 kernels. After clustering, classification, and target masking, the final mask contained 100 spatially separate connected components, matching the number of kernels. No independent per-kernel reference labels were available, so this result was not treated as an accuracy estimate. NDMI was used to demonstrate configurable overlay computation.

**Turfgrass Sample A (traffic stress, VNIR, Outdoor):** Outdoor traffic-test images were divided into three rectangular ROIs per image. Following green-leaf segmentation, raw vegetation-index counts were compared across measurement dates.

**Turfgrass Sample B (NaCl treatment, VNIR, Indoor):** Indoor images of NaCl-treated turfgrass contained five pots, each defined as a separate rectangular ROI. The resulting masks visualized the remaining photosynthetically active leaves, and raw vegetation-index frequency counts were compared between measurement dates for each pot.

These datasets illustrate the intended use of the workflow and are not independent accuracy benchmarks. In a separate VNIR turfgrass study using the same core extraction sequence, comparison with 10,000 manually labeled points yielded an overall accuracy of 0.9697, precision of 0.8830, recall of 0.9240, and F1-score of 0.9030 [12]. These values apply to that published VNIR application and do not directly validate the three demonstrations in this article.

## Limitations

1. **File format dependency:** Input loading was verified for ENVI-format data. Other formats require conversion or a loader that preserves axis order, band-center wavelengths, and reflectance calibration.
2. **Operating system:** The current implementation was tested on Windows 10/11. Compatibility with Linux and macOS has not been validated.
3. **Per-ROI processing:** Step 2 fits one ROI at a time. A saved scaler and classifier can be applied sequentially only to spectrally comparable ROIs under the conditions defined in Step 2.5 and after the transferred result has been checked. The current functions do not automate batch or concurrent execution.
4. **Manual ROI drawing:** Manual ROI coordinates may vary slightly among operators. However, Step 1 does not require pixel-accurate vegetation segmentation: as shown in Fig. 1a–c, the ROI only needs to fully enclose the target specimen and may include some surrounding background because pixel-level separation is performed in Steps 2 and 3. Thus, drawing each ROI is a straightforward coarse-cropping task rather than laborious manual segmentation. For comparisons, record the coordinates, apply the same broad delineation rule, and inspect the selected areas. Excessive unrelated background should nevertheless be avoided. The practical limitation is the time required to repeat this simple cropping operation across many units, rather than difficulty in tracing vegetation boundaries.
5. **Sensor transfer:** For a different sensor, the wavelengths required by the selected index must fall within the measured range. Changes in band number or order, spectral sampling, or radiometric preprocessing require parameter recalibration and refitting of the scaler and classifier. Possible extensions include semi-automated ROI selection, batch processing, and testing on additional file formats and operating systems.

## Ethics statements

Not applicable. This study did not involve human subjects or animal experimentation.

## Related research article

J.G. Jung, E.S. Jeong, J.Y. Jeong, J.H. Yoon, D. Shim, E.J. Bae, Hyperspectral imaging-based evaluation of seasonal growth characteristics in turfgrass, Plants 15 (2026) 1393. https://doi.org/10.3390/plants15091393.

## CRediT authorship contribution statement

**Jae Gyeong Jung:** Conceptualization, Methodology, Software, Validation, Formal analysis, Investigation, Writing – original draft, Writing – review & editing, Visualization, Data curation. **Kyuchan Kim:** Conceptualization, Methodology, Software, Validation, Investigation. **Jae Yeob Jeong:** Validation, Writing – review & editing. **Jun Hyuck Yoon:** Validation, Writing – review & editing. **Eun Ji Bae:** Conceptualization, Methodology, Validation, Resources, Writing – review & editing, Supervision, Project administration, Funding acquisition.

## Declaration of competing interest

The authors declare that they have no known competing financial interests or personal relationships that could have appeared to influence the work reported in this paper.

## Data Availability

Data will be made available on request.

## References

[1] McInnes L., Healy J., Saul N., Großberger L. (2018). UMAP: uniform manifold approximation and projection. J. Open Source Softw..

[2] Ester M., Kriegel H.-P., Sander J., Xu X., Simoudis E., Han J., Fayyad U.M. (1996). Proceedings of the Second International Conference on Knowledge Discovery and Data Mining (KDD-96).

[3] Breiman L. (2001). Random forests. Mach. Learn..

[4] Behmann J., Acebron K., Emin D., Bennertz S., Matsubara S., Thomas S., Bohnenkamp D., Kuska M.T., Jussila J., Salo H., Mahlein A.-K., Rascher U., Specim I.Q. (2018). Evaluation of a new, miniaturized handheld hyperspectral camera and its application for plant phenotyping and disease detection. Sensors.

[5] Ram B.G., Oduor P., Igathinathane C., Howatt K., Sun X. (2024). A systematic review of hyperspectral imaging in precision agriculture: analysis of its current state and future prospects. Comput. Electron. Agric..

[6] Weiss M., Jacob F., Duveiller G. (2020). Remote sensing for agricultural applications: a meta-review. Remote Sens. Environ..

[7] Min H.J., Qin J., Yadav P.K., Frederick Q., Burks T., Dewdney M., Baek I., Kim M. (2024). Classification of citrus leaf diseases using hyperspectral reflectance and fluorescence imaging and machine learning techniques. Horticulturae.

[8] Matese A., Prince Czarnecki J.M., Samiappan S., Moorhead R. (2024). Are unmanned aerial vehicle-based hyperspectral imaging and machine learning advancing crop science?. Trends Plant Sci..

[9] Reddy P., Guthridge K.M., Panozzo J., Ludlow E.J., Spangenberg G.C., Rochfort S.J. (2022). Near-infrared hyperspectral imaging pipelines for pasture seed quality evaluation: an overview. Sensors.

[10] Miao C., Pages A., Xu Z., Rodene E., Yang J., Schnable J.C. (2020). Semantic segmentation of Sorghum using hyperspectral data identifies genetic associations. Plant Phenomics.

[11] Poorter H., Hummel G.M., Nagel K.A., Fiorani F., von Gillhaussen P., Virnich O., Schurr U., Postma J.A., van de Zedde R., Wiese-Klinkenberg A. (2023). Pitfalls and potential of high-throughput plant phenotyping platforms. Front. Plant Sci..

[12] Jung J.G., Jeong E.S., Jeong J.Y., Yoon J.H., Shim D., Bae E.J. (2026). Hyperspectral imaging-based evaluation of seasonal growth characteristics in turfgrass. Plants.

